# Benzothiazole derivatives as anticancer agents

**DOI:** 10.1080/14756366.2019.1698036

**Published:** 2019-12-02

**Authors:** Ali Irfan, Fozia Batool, Syeda Andleeb Zahra Naqvi, Amjad Islam, Sameh M. Osman, Alessio Nocentini, Siham A. Alissa, Claudiu T. Supuran

**Affiliations:** a Department of Chemistry, University of Lahore, Sargodha, Pakistan; b College of Materials Engineering, Fujian Agriculture and Forestry University, Fuzhou, P.R. China; c Chemistry Department, College of Science, King Saud University, Riyadh, Saudi Arabia; d NEUROFARBA Department, Section of Pharmaceutical and Nutraceutical Sciences, University of Florence, Sesto Fiorentino (Firenze), Italy; e Chemistry Department, College of Science, Princess Nourah bint Abdulrahman University, Riyadh, Saudi Arabia

**Keywords:** Benzothiazole, anticancer agent, drug targets, scaffold, carbonic anhydrase inhibitor

## Abstract

Benzothiazole (BTA) belongs to the heterocyclic class of bicyclic compounds. BTA derivatives possesses broad spectrum biological activities such as anticancer, antioxidant, anti-inflammatory, anti-tumour, antiviral, antibacterial, anti-proliferative, anti-diabetic, anti-convulsant, analgesic, anti-tubercular, antimalarial, anti-leishmanial, anti-histaminic and anti-fungal among others. The BTA scaffolds showed a crucial role in the inhibition of the metalloenzyme carbonic anhydrase (CA). In this review an extensive literature survey over the last decade discloses the role of BTA derivatives mainly as anticancer agents. Such compounds are effective against various types of cancer cell lines through a multitude of mechanisms, some of which are poorly studied or understood. The inhibition of tumour associated CAs by BTA derivatives is on the other hand better investigated and such compounds may serve as anticancer leads for the development of agents effective against hypoxic tumours.

## 1. Introduction

Heterocyles are important pharmcophores and have significance to create privileged chemical structures possessing pharmacological activities. Five membered heterocyclic which incorporate oxygen, nitrogen and sulphur are found in broad spectrum therapeutic agents which have an enormous significance in drug discovery and drug development processes^1^. Benzothaizole (BTA) is a fused benzoheterocyle which is present in many naturally occurring products and is responsible for the medicinal, pharmacological and pharmaceutical applications of such natural products^2^. BTA is present in terrestrial as well as marine compounds which exhibit various biological activities^3^. The BTA nucleus is formed by the fusion of the thiazole ring with a benzene ring^4^.

**Figure 1.**
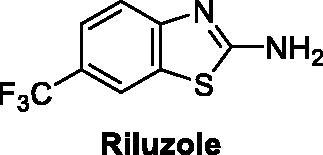
Structure of riluzole, a drug for amyotrophic lateral sclerosis.

The pharmacological profile of the drug used for the management of amyotrophic lateral sclerosis Riluzole (Figure 1) attracted the attention of medicinal chemists towards biologically active benzothiazole^5^.

The BTA scaffold possesses a wide spectrum of biological activities such as anti-inflammatory^6^, fungicidal^7^, anti-diabetic^8^, analgesic^9^, anti-microbial^10^, antitumor^11^, antileishmanial^12^, antheltmintic^13^, antirheumatic^14^ and CNS depressant^15^ etc. BTA derivatives exhibit remarkable and prevalent biological and pharmacological activities against different types of tumours and cancer cell lines such as HeLa (human cervical cancer cell line), SW480 (human colon adenocarcinoma cell line), HepG2 (human liver carcinoma cells)^16^, mammary and ovarian tumour cell lines^17^, colon, nonsmall-cell lung and breast subpanels cell lines^18^, and HCC (hepatocellular carcinoma)^19^ etc.

Cancer is the most prominent, notably complex and lethal disease which became a serious concern of today’s medical science. It poses a great challenge to medical scientific community for development of drugs, medicines and procedures for safer treatment and cure of cancer disease^20^. These neoplasm tumour cells are diversified, heterogeneous cells with rapid proliferative properties. These neoplasm malignant tumours, have potential to invade or spread to other parts of body through blood stream and lymphatic system^21^. The plethora of research mentioned in the present review of last decade on anticancer potential of BTA derivatives will be helpful in future drug discovery and drug development for the treatment of lethal cancer disease.

## 2. BTA derivatives as anticancer agents

### 2.1. Fluorinated derivatives of benzothiazole as anticancer agents

**Figure 2.**
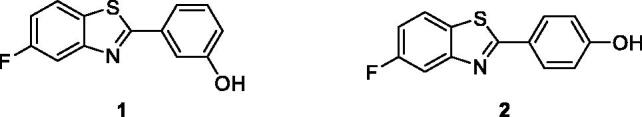
Substituted phenols containing a flourobenzothiazol scaffold **1** and **2**.

**Figure 3.**
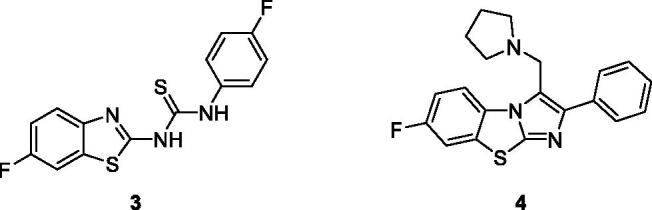
Substituted thiourea containing benzothiazole derivative **3** and the **s**ubstituted pyrrolidine based imidazo benzothiazole derivative **4**.

Aiello et al. synthesised fluorinated 2-aryl benzothiazole derivatives and evaluate them for anti-tumour activities against cancer cell lines such as MDA-MB-468 (mammary gland/breast tissues derived from metastatic site) and MCF-7 cell line (human breast adenocarcinoma). The fluorinated BTA derivatives **1** (3–(5-fluorobenzo[d]thiazol-2-yl)phenol) and **2** (4–(5-fluorobenzo[d]thiazol-2-yl)phenol) having hydroxyl substituents on the third and fourth position of phenyl exhibited the best activity having GI_50_ values of 0.57 and 0.4 µM respectively against MCF-cell line as compared to BTA derivatives containing alkoxy, methyl sulphonyl and ethyl substituents on the benzothiazole (Figure 2). Kumbhare et al. afforded the *N*-bis-benzothiazole and benzothiazolyl thiocarbamide derivatives and screened for cytotoxic activities against two human cell lines U-937 (human macrophage cell line), THP-1 (human leukaemia monocytic cell line) and B16-F10 (mouse melanoma cell line). The thiourea containing benzothiazole derivative **3** (Figure 3) demonstrated the best antiproliferative activity against the U-937 cell line as compared to standard drug Etoposide. The IC_50_ values of compound **3** were higher (16.23 ± 0.81 µM)), (4847.73 ± 2.39 µM)) and (34.58 ± 1.73 µM)) as compared to standard compound etoposide IC_50_ values (17.94 ± 0.89), (18.69 ± 0.94) and (2.16 ± 0.11 µM)) against U-937, B16-F10 and THP-1 cell lines respectively^22^.

Kumbhare et al. reported the synthesis of mannich base arylimidazo derivatives containing benzothiazole moiety and screened for their anticancer activities against HepG2, MCF -7 and HeLa cell lines. All these synthesised mannich bases BTA scaffolds showed cytotoxicity against all tested cell lines but the pyrrolidine based imidazo benzothiazole derivative **4** (Figure 3) demonstrated specific features of apoptosis as enhancement in the levels of caspase-3. The compound **4** exhibited anti cancer activity and proved to be the best antiproliferative agent as compared to other derivatives against HepG2, MCF-7 and HeLa cell line when screened at 4.0 µM concentrations. The SAR studies revealed that the incorporation of fluorine atom at the 7^th^ position of derivative **4** enhanced the cytotoxicity. The compound **4** have potential to lead in the treatment of cancer especially against hepatocaricinoma. The anticancer activity potential of BTA scaffold **4** is encourging for the development of new anti-cancer therapeutic agents and this will be good addition in armamentarium that consists of paclitaxel, cisplatin and doxorubicin drugs^23^.

**Figure 4.**
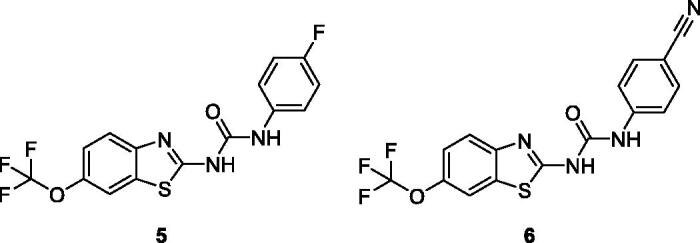
Substituted fluorophenyl containing benzothiazole urea derivative **5** and 2-substituted cyanophenyl containing benzothiazole urea derivatives **6**.

Caputo et al. afforded two types of five derivatives on the basis of an aryl amide and an aryl urea functionalities attached at C-2 of benzothiazole core and these scaffolds were screened against 60 human cancer cell lines. The urea moiety based fluorophenyl containing benzothiazole derivative **4** (Figure 3) and cyanophenyl containing benzothiazole derivative **5** (Figure 4) demonstrated remarkable anticancer activities. The BTA derivative **4** exhibited the anticancer activity at 10^−5 ^M against different cell lines such as leukaemia cell lines (log GI_50_ value −5.48), non-small cell lung cell lines (log GI_50_ value −5.48), colon cancer cell lines (log GI_50_ value −5.51), central nervous system cancer cell lines (log GI_50_ value −5.49), melanoma cell lines (log GI_50_ value −5.48), ovarian cancer cell lines (log GI_50_ value −5.49), renal cancer cell lines (log GI_50_ value −5.53), prostate cancer cell lines (log GI_50_ value −5.50) and breast cancer cell lines (log GI_50_ value −5.56) in comparison with reference drug 5-fluorouracil NSC 19893. The BTA scaffold **5** showed remarkable growth inhibitory activities against different human tumour cell lines such as leukaemia cell lines (log GI_50_ value −5.93), non-small cell lung cell lines (log GI_50_ value −6.0), colon cancer cell lines (log GI_50_ value −5.89), central nervous system cancer cell lines (log GI_50_ value −5.73), melanoma cell lines (log GI_50_ value −5.89), ovarian cancer cell lines (log GI_50_ value −5.74), renal cancer cell lines (log GI_50_ value −5.90), prostate cancer cell lines (log GI_50_ value −5.72) and breast cancer cell lines (log GI_50_ value −6.0) as compared with reference drug 5-fluorouracil NSC 19893. The scaffolds **4** and **5** showed the best anticancer therapeutic potential due to presence of electron with drawing groups on *para* position of phenyl ring^23^.

**Figure 5.**
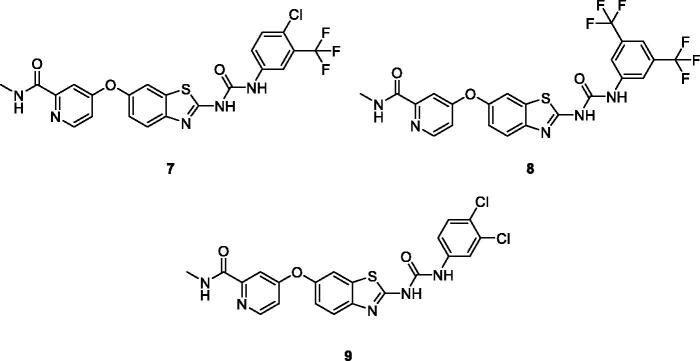
Derivative **7**–**9** discussed in the paper.

El-Damasy et al. synthesised the novel amide and urea based BTA series of 20 sorafenib analogues in which the pyridylamide privileged functionality was attached with an ether linkage at 6-position of the BTA ring. A selected group of 12 potent scaffolds were evaluated and appraised for anti-proliferative activities against sixty human cancer cell lines. These chlorotrifluoromethyl phenyl ureido picolinamide benzothiazoles **7** (Figure 5), bis-trifluoromethyl phenyl ureido based picolinamide benzothiazole derivative **8** (Figure 5) and dichlorophenyl ureido based picolinamide benzothiazoles **9** (Figure 5) were more potent in the treatment of renal cell carcinoma than the standard drug sorafenib, used for the treatment of such tumours. The 3,5-bis-trifluoromethylphenylurea **8** showed good inhibitory activities against ACHN (renal cancer cells lines) and A-498 (human kidney carcinoma cell line) with GI_50_ values of 0.542 µM and 1.02 µM respectively. This compound also possess efficacy against UO-31 and RXF 393 cell lines. The derivatives **7** and **9** because of 3,4-disubstitutedphenyl moiety, exhibited excellent anti-proliferative activities with low IG_50_ values of 1.85, 2.10 µM against RCC and ACHN cell lines respectively. The SAR study revealed the fact that sorafenib analogues possesses anti proliferative activity due to the presence of both urea spacer and phenyl disubstitution. Compound **8** demonstrated the highest CLogP value being the most lipophilic and potent derivative, in the low µM range^24^.

**Figure 6.**
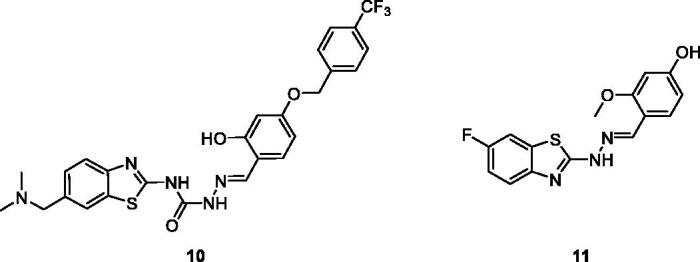
Benzothiazoles **10** and **11**.

Ma et al. reported BTA derivatives containing an *ortho*-hydroxy-N-acyl hydrazone moiety for antiproliferative activities and procaspase-3 kinase activation activities against five different cell lines, namely MDA-MB-231 (human breast adenocarcinoma cell line), MNK-45 (gastric cancer cell line), NCI-H226 (human lung cancer cell line), HT-29 (human colorectal adenocarcinoma cell line) and SK-N-SH (neuroblastoma cell line). The substituted 2-hydroxybenzylidene containing semicarbazide **10** (Figure 6) showed inhibitory activities against all cell lines with IC_50_ and EC_50_ values ranging from 0.24 to 0.92 µM and 0.31 µM respectively. The SAR studies revealed the paharmacological activities of BTA scaffold **10** in *in-vitro* is due to introduction of phenyl and benzyloxyl substitutions^25^.

Gabr et al. obtained hydrazine derivatives by treating 2-amino-6-fluorobenzothiazole with hydrazine hydrate which was further treated with the suitable aldehydes to afford 27 different BTA Schiff base derivatives. These derivatives were screened for anti-tumour potential against Hela (cervical cancer) and COS-7 (kidney fibroblast cancer) cell lines. The hydrazine based benzothiazole **11** (Figure 6) exhibited IC_50_ of 2.41 µM and 4.31 µM against Hela and COS-7 cell lines as compared to reference doxorubicin having IC_50_ 2.05 µM and 3.04 µM respectively. The SAR studies explained the effect of various substitutions on activities of all the synthesised derivatives. The scaffold present in **11** has the 2–(4-hydroxy-methoxy benzylidene)-hydrazino moiety at the C-2 position which remarkably enhances the anti-tumour potential, whereas replacing the 4-hydroxy moiety with 4-methoxy decreased the activities against both cell lines^26^.

**Figure 7.**
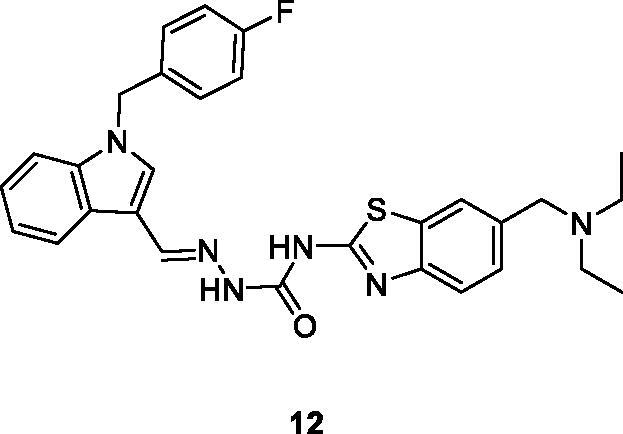
Indol based hydrazine carboxamide benzothiazole derivative **12**.

Junjie et al. reported the synthesis of semicarbazone containing BTA derivatives by the reaction of 4-nitrobenzyl bromide with substituted amines under different reaction conditions and evaluated their anticancer activity against four different cancer cell line such as human colon cancer cells (HT29), human lung cancer cell (H460), non-small cell lung cancer (A549) and human breast cancer (MDA-MB-231). Among these derivatives, the indole based hydrazine carboxamide scaffold **12** (Figure 7) showed potent antitumor activity with IC_50_ values of 0.015 µM for HT29, 0.28 µM for H460, 1.53 µM for A549 and 0.68 µM for MDA-MB-231. The structure – activity relationship explained that compound **12** exhibited the highest antitumor activity due to the presence of electron withdrawing groups in the 4 position of benzyl ring^27^.

### 2.2. Imidazole based benzothiazole derivatives as anticancer agents

**Figure 8.**
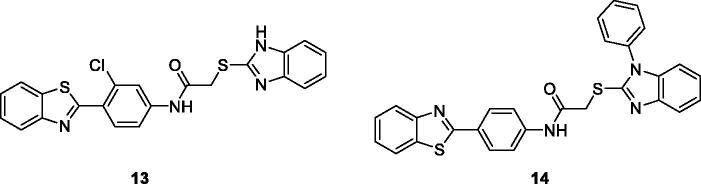
Substituted phenyl imidazole based benzothiazoles **13** and **14**.

Yurttas et al. obtained 2–(4-aminophenyl)BTA derivatives substituted with different heterocyclic rings and tested their antitumor potential against 60 human tumour cell lines. The BTA derivatives **13** (2–(1*H*-benzo[d]imidazol-2-ylthio)-*N*-(4-(benzo[d]thiazol-2-yl)-3-chlorophenyl) acetamide) (Figure 8) and **14** (*N*-(4-(benzo[d]thiazol-2-yl)phenyl)-2-(1-phenyl-1*H*-benzo[*d]*imidazol-2-yl-thio)-acetamide) (Figure 8) showed remarkable antitumor potential against different cancer cell lines. The heterocylic substitutions affect the activity and antitumor potential of these BTA derivatives, with derivative **14** having comparable antitumor potential with the standard drugs whereas derivative **13** being less active compared to **14**. The order overall antitumor potential of 2–(4-aminophenyl) benzothiazole derivatives with reference to the heterocyclic substitution was benzimidazole ≥ imidazole > benzothiazole > benzoxazole^28^.

**Figure 9.**
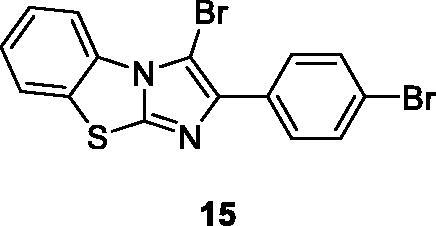
Imidazole based benzothiazole derivative **15**.

Singh et al. reported the synthesis of imidazole based benzothiazoles by treatment of substituted anilines with KSCN which afforded the desired benzothiazole derivatives, and studied their anticancer activities. Compound **15** (Figure 9) showed excellent anticancer activity possessing IC_50_ value 10 µM when compared with the standard drug doxorubicin^29^.

### 2.3. Piperazine based benzothiazole derivatives as anticancer agents

**Figure 10.**
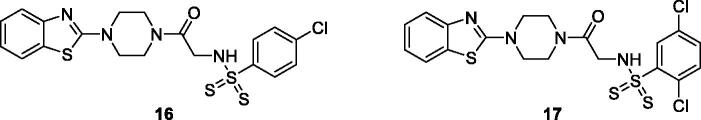
Di-/monochlorobenzenesulfonamide based piperazine benzothiazoles derivative **16** and **17**.

Al-Soud. et al. reported the synthesis of BTA derivatives incorporating sulphonamide, piperazino-arylsulfonamide and arylthiol scaffolds and determined their anti-proliferative potential against different cell lines, such as CCRF-SB (Human acute B-lymphoblastic leukaemia), DU-145 (human prostate cancer cell lines express androgen receptor), HepG-2 cell line (human liver cancer), WIL-2NS (Human splenic B-lymphoblastoid cells), MRC-5 (human lung fibroblast cell line), MCF-7 cell line (human breast adenocarcinoma), MT-4 (human T-cells containing an integrated HTLV-1 genome), SK-MES-1 cell line (human lung cancer) and SK-28 cell line (skin melanoma). Derivative **16** (*N*-(2–(4-(benzo[d]thiazol-2-yl)piperazin-1-yl)-2-oxoethyl)-4-chloro-benzenesulfonodithioamide) showed antiproliferative activity (CC_50_ = 8 ± 3 µM) against human derived DU-145 cell line (Figure 10) whereas derivative **17** (*N*-(2–(4-(benzo[d]thiazol-2-yl) piperazin-1-yl)-2-oxoethyl)-2,5-dichloro benzenesulfonodithio-amide) demonstrated remarkable activities against several human derived cell lines such as HepG2 and DU-145 (with CC_50_ of 8 ± 2 µM, and 9 ± 2 µM, respectively) (Figure 10). Derivatives **16** and **17** exhibited atiproliferative potential due to the introduction of chloro and dichloro phenyl groups while their replacement of with hydrogen, methoxy, nitro, triflouromethyl and methyl groups lead to a decrease in the antiproliferative potential of BTA derivatives^30^.

**Figure 11.**
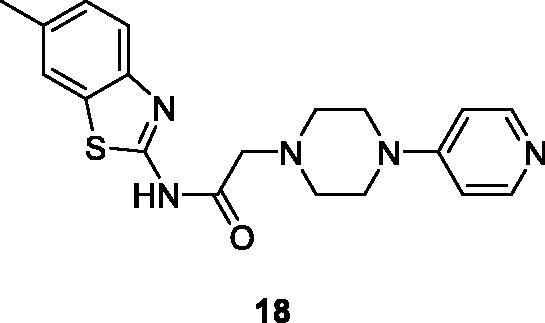
Pyridine conataining piperazine benzothiazole derivative **18**.

Gurdal et al. synthesised BTA derivatives which incorporate piperazine moieties and evaluated their cytotoxicity against different cancer cell lines such as HUH-7 (Heptacellular), MCF-7 (Breast) and HCT-116 (Colorectal). GI_50_ values of these derivatives indicated that all compounds exhibited good potential against the aforementioned cell lines but the pyridine containing derivative **18** (Figure 11) had a remarkable cytotoxic activity with GI_50_ value 7.9 µM, 9.2 µM and 3.1 µM for HCT-116, MCF-7 and HUH-7 respectively. Apoptosis caused by this derivative during cell cycle arrest at subG1 phase was confirmed by Hoechst staining and fluorescence activated cell sorting analysis^31^.

### 2.4. Oxadiazole based benzothiazole derivatives as anticancer agents

**Figure 12.**
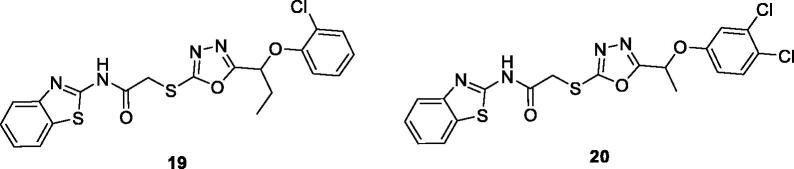
Oxadiazole based acetamide benzothiazole derivatives **19** and **20**.

Akhtar et al. synthesised BTA and 1,3,4-oxadiazole-2-thione derivatives and determined their antitumor potential in *in-vitro* against different tumour cell lines. BTA derivatives **19** (N-(benzo[d]thiazol-2-yl)-2–(5-(1–(2-chlorophenoxy)propyl)-1,3,4-oxadiazol-2-ylthio)acetamide) and **20** (N-(benzo[d]thiazol-2-yl)-2–(5-(1–(3,4-dichlorophenoxy)ethyl)-1,3,4-oxadiazol-2-ylthio) acetamide) exhibited remarkable activities against CCRF-CEM (leukaemia) cell lines. The CC_50_ values of compounds **19** and **20** (CC_50_ = 12 ± 2 µM and 8 ± 1 µM respectively) were comparable to the standard drug Doxorubicin. The replacement of the chloro moiety with bromo in the hybrid structures of **19** and **20** decreased their anti-tumour potential (Figure 12)^32^.

### 2.5. Morpholine-thiourea based benzothiazole derivatives as anticancer agents

**Figure 13.**
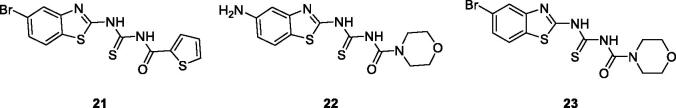
Morpholine based thiourea bromobenzothiazoles **22–24**.

Saeed et al. reported the synthesis of benzothiazole moiety based thiourea derivatives. These novel derivatives were tested for anticancer potential against cancer cell lines MCF-7 and HeLa cells. The MTT assay (colorimetric assessment of cell metabolic activity) indicated that the thiophene based acetamide benzothiazole derivatives **21** (Figure 13), morpholine based thiourea aminobenzothiazole derivative **22** (Figure 13) and morpholine based thiourea bromobenzothiazole **23** (Figures 13) were potent anticancer agents having IC_50_ values of 24.15, 26.43 and 18.10 µM against MCF-7 cell line, and of 46.46, 45.29 and 38.85 µM against HeLa cells lines respectively^33^.

**Figure 14.**
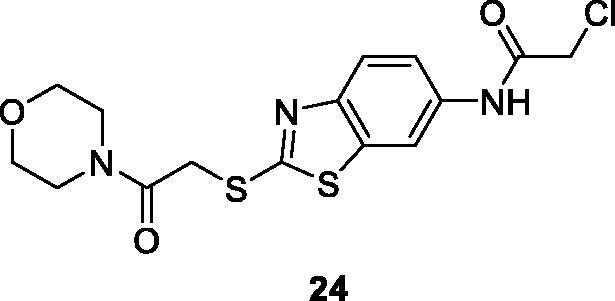
Morpholine based acetamide benzothiazole **24**.

Lei et al. obtained the morpholine based acetamide benzothiazole derivative **24** (Figure 14) by the treatment of morpholine with 2-chloroacetyl chloride and studied its anticancer activity against HCC (human hepatocellular carcinoma) cell lines HepG2 and Bel7402, reporting IC_50_ value for HepG2 and Bel7402 in the millimolar range^34^.^.^

### 2.6. Thiophene based benzothiazole derivatives as anticancer agents

**Figure 15.**
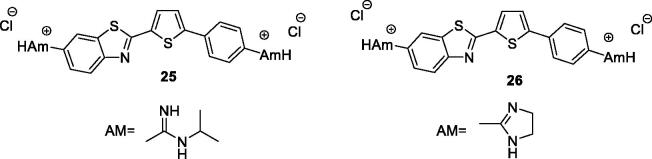
Imidazolinyl-substituted thiophene-based benzohiazoles **25** and **26**.

Racane et al. obtained the furyl and thienyl based diamidino substituted derivatives of phenyl-BTA and screened them for antiproliferative activities against different tumour cell line *in vitro*. Derivative **25** (diamidino-substituted thiophene based BTA, Figure 15) and **26** (imidazolinyl-substituted thiophene based BTA; Figure 15) exhibited low cytotoxic effects on normal human fibroblasts and strong antiproliferative effects on MiaPaCa-2 and MCF-7 cancer cell lines. The experimental data indicated that the benzothiazole derivatives having thiophene and imidazole substitions possessed interesting antiproliferative activities^35^.

### 2.7. Thiadiazole based benzothiazole derivatives as anticancer agents

**Figure 16.**
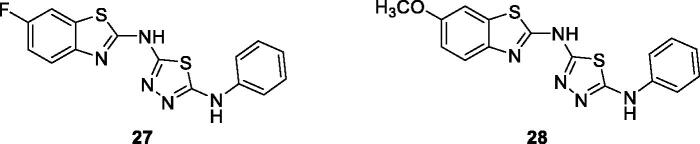
Substituted thiadiazole based BTAs **27** and **28**.

Sekar et al. reported the synthesis and anticancer activities of six noval BTA derivatives. All derivatives exhibited anticancer activities from a high to a moderate activity level with the substituted thiadiazole flourobenzothiazole **27** (Figure 16) and methoxybenzothiazole **28** (Figure 16) showing the best anti-cancer potential due to the presence of highly electronative and electron denoting pharmacophores (e.g. fluorine and methoxy moieties)^36^.

### 2.8. Substituted pyridine based benzothiazole derivatives as anticancer agents

**Figure 17.**
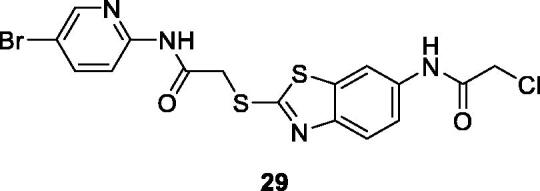
Substituted bromopyridine based acetamide benzothiazole derivative **29**.

Shi et al. synthesised 20 BTA-2-thiol derivatives and investigated their anti-tumour potential against different cell lines such as SW480 (colon adenocarcinoma), HeLa, A549, HCT-116, HepG2 and SKRB-3 breast cancer cell line. The substituted bromopyridine acetamide benzothiazole derivative **29** (Figure 17) showed potent antitumor activity against SKRB-3, SW620, A549 and HepG2 cell lines with IC_50_ values of 1.2 nM, 4.3 nM, 44 nM and 48 nM, respectively. Apoptosis was the mechanism of cell death and was concentration dependent in HepaG2 cells. These results indicated that BTA-2-thiols exhibit broad spectrum anti-cancer activities which are worth to be further investigated^16^.

**Figure 18.**
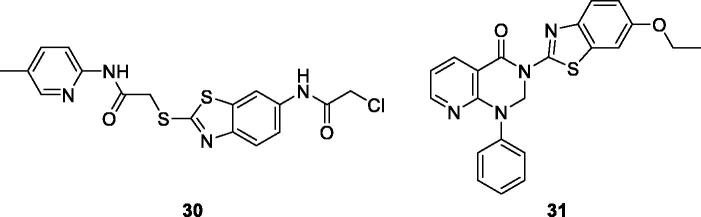
Substituted pyridine based acetamide benzothiazole **30** and pyridine containing pyrimidine benzothiazole **31**.

Xuejiao et al. reported the synthesis of a substituted pyridine based acetamide BTA derivative **30** (Figure 18) and screened its anti-cancer activity both *in vitro* and *in* v*ivo.* Derivative **30** demonstrated anti-proliferative activities against a wide spectrum of human cell lines and induced the mitochondrial apoptotic pathway in HepaG2 cell lines. The BTA scaffold **30** proved to be a promising candidate for cancer chemotherapy^37^.

Kamal et al. reported the synthesis of novel phenyl pyridopyrimidinones based BTA derivatives which were examined against four different cancer cell lines such as ME-180, DU-145, MCF-7 and B-16. The pyridine containing pyrimidine benzothiazole **31** (Figure 18) exhibited interesting cytotoxicity with IC_50_ value of 4.01 µM against ME-180 (human cervical cancer cell line)^38^.

### 2.9. Pyrazole based benzothiazole derivatives as anticancer agents

**Figure 19.**
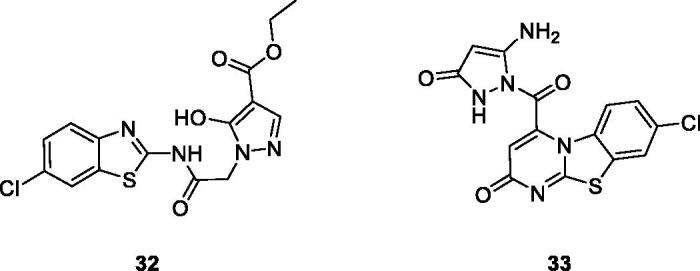
Pyrazole based benzothiazoles **32** and **33**.

Gabr et al. reported the synthesis and evaluation of novel BTA scaffolds against 60 tumour cell lines at a single dose of 10 µM. The best derivatives were **32** (Figure 19) and **33** (Figure 19), which were further screened at 5 doses. These derivatives demonstrated interesting anticancer activity at micro molar and sub micro molar concentrations, against all sixty tumour cell lines with GI_50_ in the low micro molar or submicromolar range. The SAR study revealed that introduction of the pyrazole moiety significantly enhanced the antitumor activity of both derivatives. Furthermore the presence of 2-hydroxy ester and 3-oxopyrazole within the pyrimidine moiety increased the anti-tumour activities of both derivatives. The simple BTA scafolds having pyrazole functionalities were more potent against different cell lines as compared to derivatives having the pyrazole ring within a pyrimidine moiety^39^.

### 2.10. Pyrimidine based benzothiazole derivatives as anticancer agents

**Figure 20.**
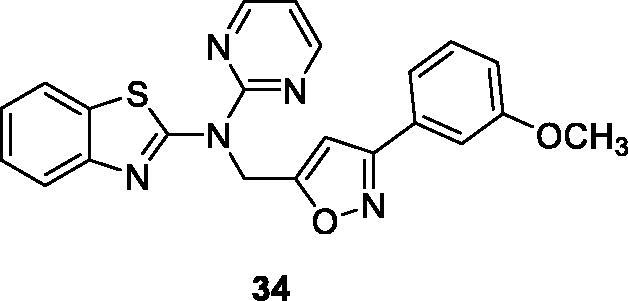
Structure of pyrimidine based isoxazole derivative **34**.

Kambhare et al. synthesised the isoxazole pyrimidine based BTAs and evaluated them for anticancer activity by the MTT assay against different cell lines such as A549, Colo205, MCF-7 and U937 cell lines, in comparison with the standard drug etoposide. The pyridine containing pyrimidine derivative **34** (Figure 20) demonstrated good anti-cancer potential with IC_50_ value 5.04 µM against colo205, 13.9 µM against U937, 30.67 µM against MCF-7 and 30.45 µM against A549 cell lines when compared with standard drug etoposide. The scaffold **34** activated p53 or TP53 (tumour protein) pathways, which regulate the equilibrium between apoptosis and cell proliferation. The SAR explained that the maximum cytotoxicity of derivative **34** against colon cancer cell line was due to the presence of methoxy group (–OCH_3_) in the phenyl^40^.

**Figure 21.**
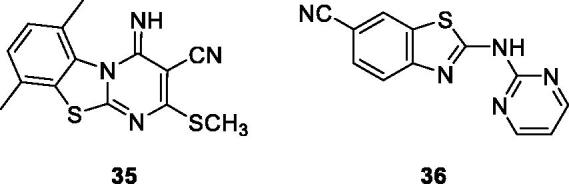
Pyrimidine based benzothiazole derivative **35** and **36**.

Waghmare et al. reported the synthesis of substituted pyrimidine containing benzothiazole derivative **35** (Figure 21) by refluxing BTAs with bis-methylthio methylene malononitrile, and tested their anticancer activity against 18 different cell lines. The scaffold **35** possessed excellent anticancer activity with a good percentage of growth inhibition against lung cancer, breast cancer and renal cancer cell lines. The good anticancer activity of derivative **35** is due to the presence of two methyl and one SCH_3_ groups in its structure^41^.

Caleta et al. synthesised cyano and amidinobenzothiazole substituted anilins which were treated with 2-bromo-6-cyanobenzothiazole under specific reaction conditions to afford compounds which have been studied for their anticancer activity against 6 cancer cell lines such as laryngeal carcinoma (Hep-2), breast carcinoma (MCF-7), cervical carcinoma (HeLa), pancreatic carcinoma (MiaPaCa-2), colon carcinoma (SW 620), lung carcinoma (H 460), and diploid fibroblasts (WI 38). Among them, the pyrimidine based carbonitrile benzothiazole derivative **36** (Figure 21) showed potent activity against all cancer cell lines used in the study^42^.

### 2.11. Piperidine based benzothiazole derivatives as anticancer agents

**Figure 22.**
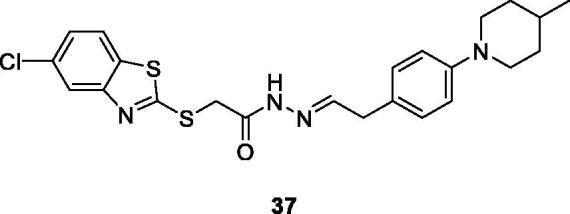
Piperidine based acetohydrazide benzothiazole **37**.

Osmaniye et al. prepared BTA acylhydrazone using 4-fluorobenzaldehyde refluxed with substituted amines, to afford the desired derivatives and studied their anticancer activities against rat brain glioma (carcinogenic C6) cell line, human lung adenocarcinoma epithelial (A549) cell line, human breast adenocarcinoma (MCF-7) cell line, human colorectal adenocarcinoma (HT-29) cell line and mouse embryo fibroblast (NIH3T3) cell line. The piperidine based acetohydrazide derivative **37** (Figure 22) showed modest activity having IC_50_ value 1 < mM, 0.03 mM, 0.10 mM, 0.30 mM and 1< mM for A549, HT-29, MCF-7, C6, and NIH3T3 cell lines respectively against reference drug cisplatin^43^.

### 2.12. Secondary sulphonamide benzothiazole derivatives as anticancer agents

**Figure 23.**
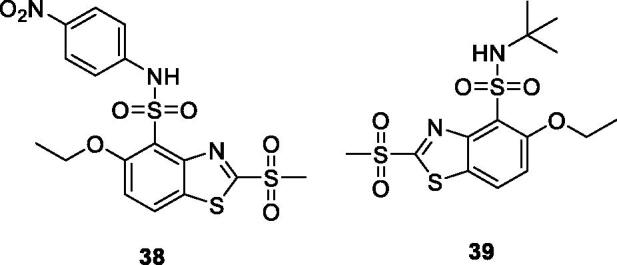
Sulphonamide based methylsulfonyl benzothiazoles **38** and **39**.

Lad et al. reported the synthesis of a series of methylsulfonyl benzothiazoles, obtained from 5-ethoxybenzothiazol-2-amine which were tested their anticancer activities. Among these derivatives, the nitrophenyl sulphonamide based methylsulfonyl benzothiazole **38** (Figure 23) and *ter*-butyl sulphonamide based methylsulfonyl benzothiazole **39** (Figure 23) exhibited the best anticancer activities against HeLa cell line with the IG_50_ value of 0.22 µM and 0.6 µM respectively^44^.

**Figure 24.**
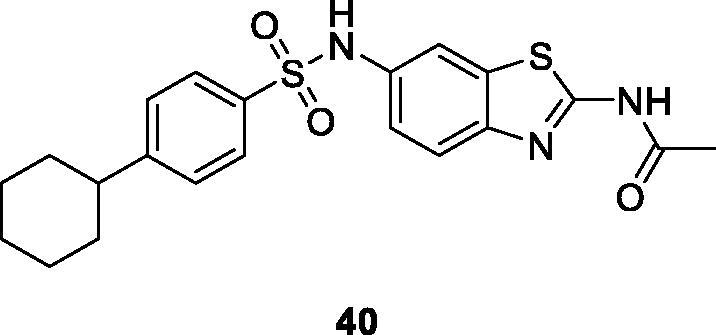
Secondary sulphonamide based acetamide benzothiazole **40**.

Sadhasivam et al. reported the synthesis of 2, 6-disubstituted-BTA by reaction of 2-amino-6-nitrobenzothiazole and acetic anhydride and studied their anticancer activity against three cancer cell lines MCF-7, HeLa and MG63 (human osteosarcoma) The sulphonamide scaffold based BTA **40** (Figure 24) exhibited modest anti-cancer activity with IC_50_ of 34.5 µM for MCF-7, 44.15 µM for HeLa and 36.1 µM for the MG63^45^.

### 2.13. Benzamide based benzothiazole derivatives as anticancer agents

**Figure 25.**
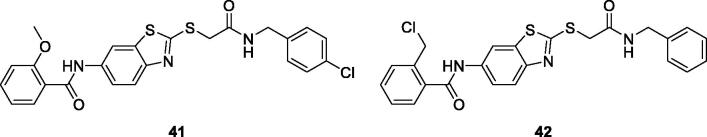
Substituted methoxybenzamide based benzothiazole derivative **41** and chloromethylbenzamide based benzothiazole **42**.

Wang et al. synthesised and evaluated 24 benzothiazole-2-thiol derivatives for antiproliferative activities against different human cancer cell lines such as A549, HCT-116, SW620, SW480, MDA-MB-468, SKRB-3, HeLa, SKOV-3, PC-3, BxPC-3, A431 and A375. Some derivatives exhibited better anticancer activities as compared to the standard drug cisplatin. The substituted methoxybenzamide benzothiazole **41** and the substituted chloromethylbenzamide benzothiazole **42** (Figure 25) showed good anti-tumour potential *in vitro*, with IC_50_ values ranging from 1.1 µM to 8.8 µM. The introduction of chloromethyl and methoxy functionalities in compounds **41** and **42** increased their anticancer activity compared to other synthesised analogs^46^.

**Figure 26.**
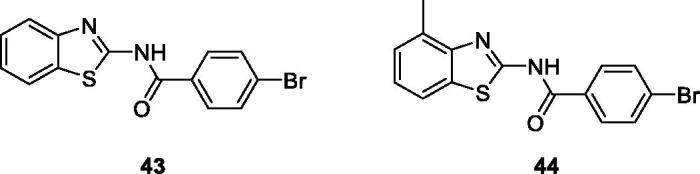
Benzamide containing benzothiazoles **43** and **44**.

Bolelli et al. synthesised 2-substituted benzothiazoles by the reaction of carboxylic acid with thionyl chloride which afforded acyl chlorides, further treated with substituted benzothiazole to give 2-substituted benzothiazoles, which were studied for their inhibitory activities against human glutathione transferases (hGSTP1-1). The benzamide benzothiazole derivative **43** and benzamide methylbenzothiazole **44** (Figure 26) showed potent hGSTP1-1 inhibitory activities, useful in cancer chemotherapy. The SAR studies pinpointed that both these scaffold showed effective inhibition due to the presence of *para*-substitutions on the phenyl ring of the benzamide group^47^.

**Figure 27.**
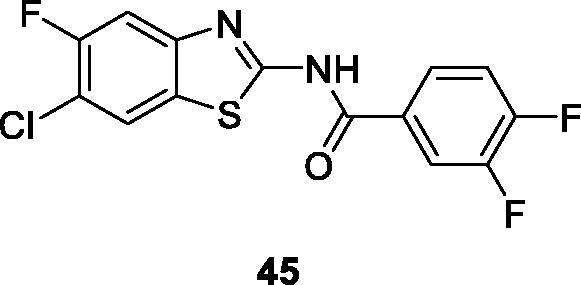
Substituted difluorobenzamide BTA derivative **45**.

Corbo et al. reported 19 derivatives of benzamide based BTAs by the reaction of substituted aniline, potassium thiocyante and bromine, which afforded substituted compounds which were tested for anti-proliferative activity against MCF-7 and HepG2 cell lines. The substituted diflourobenzamide containing benzothiazole **45** (Figure 27) proved to be a potent anti-proliferative compound with the percentual inhibition of 64 ± 2 µM for MCF-7 cell line and of 64 ± 6 µM for HepG2 cell line^48^.

### 2.14. Quinolone based benzothiazole derivatives

**Figure 28.**
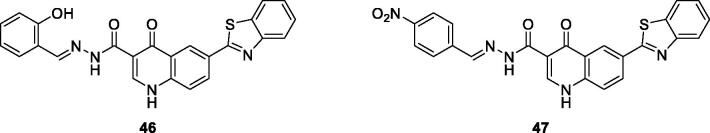
Hydroxybenzylidine containing quinolone benzothiazole derivative **46** and **the** nitrobenzylidene quinolone derivative **47**.

Abdelgawad et al. synthesised quinolone based benzothiazole derivatives by the treatment of substituted benzothiazoles with aromatic aldehydes. The nitrobenzylidene containing quinolone derivative **46** (Figure 28) and the hydroxybenzylidine containing derivative **47** (Figure 28) showed antitumor activities against the MCF-7 cell line, having IC_50_ values of 0.058 µM and 0.052 µM, respectively^49^.

**Figure 29.**
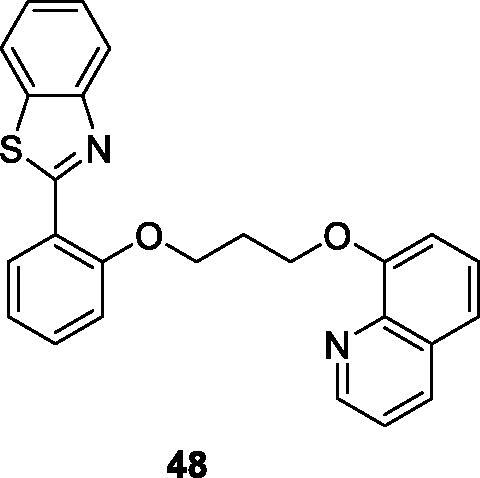
Quinolone based benzothiazole derivative **48**.

Sarkar et al. prepared benzothiazolyl quinoline derivatives by treatment of 2-aminobenzothiol with 2-hydroxy-benzaldehyde and studied their activities for the A1, A2A, A2B and A3 adenosine receptors. The A3 receptor is overexpressed in different cancer cell lines. The quinolone based derivative **48** (Figure 29) showed the maximum potency for the hA3 adenosine receptor^50^.

### 2.15. Miscellaneous benzothiazole derivatives as anticancer agents

**Figure 30.**
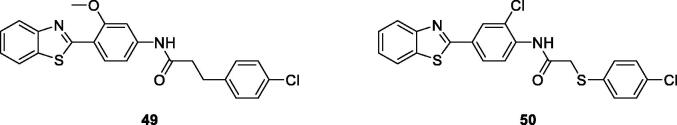
Substituted propanamide/acetamide based benzothiazoles **49** and **50**.

Tay et al. synthesised and evaluated *N*-[4-(benzothiazole-2yl) phenyl]-2-aryloxyacetamide derivatives for cytotoxicity and anticancer activity against sixty human cancer cell lines derived from 9 neoplastic diseases, among which L (leukaemia), M (melanoma), RC (renal cancer) NSCLC (non-small cell lung cancer) CC (colon cancer), OC (ovar ian cancer), BC (breast cancer), CNSC (central nervous system cancer) and PC (prostate cancer). Derivatives **49** (*N*-(4-(benzo[d]thiazol-2-yl)-3-methoxyphenyl)-3–(4-chlorophenyl)propanamide) and **50** (*N*-(4-(benzo[d]thiazol-2-yl)-2-chlorophenyl)-2–(4-chlorophenylthio)acetamide) (Figure 30) exhibited interesting anti-cancer activities. The SAR studies investigated that anticancer activities of these compounds were due to the substitutions (methoxy and chloro groups) present in their molecules^51^.

**Figure 31.**
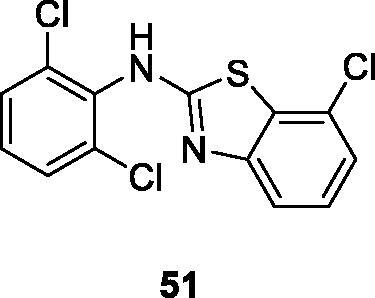
Dichlorophenyl-chlorobenzothiazole derivative **51**.

Noolvi et al. reported the synthesis of chloro substituted benzothiazole amines in order to produce isothiocyanates and thioureases. These BTA derivatives were tested for their anticancer activities. The dichlorophenyl containing chlorobenzothiazole **51** (Figure 31) showed good anticancer activity against 9 different cancer cell lines having GI_50_ values in the range of 1.60 µM–71.8 nM. The derivative **51** exhibited GI_50_ = 7.18 × 10^−8 ^M against non-small cell lung cancer (HOP-92). The SAR studies showed that the highest activity of compound **51** was due to the presence of three chlorine atoms in the compound as compared to other derivatives^52^.

**Figure 32.**
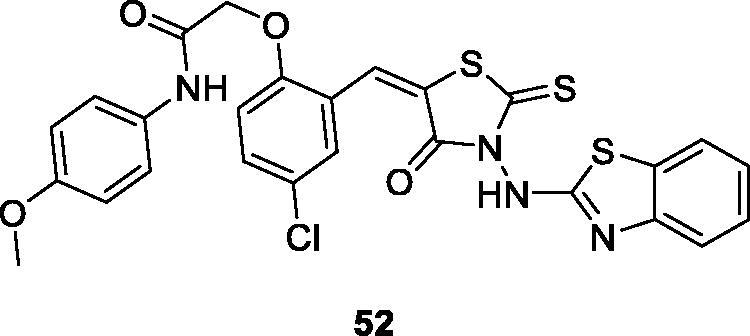
Substituted thioxothiazolidine acetamide benzothiazole derivative **52**.

Havrylyuk et al. screened novel 4-thiazolidinone benzothiazole derivatives against ovarian, renal, prostate, leukaemia, melanoma, lung, colon, CNS and breast cancer cell lines. The thioxothiazolidine acetamide benzothiazole **52** (Figure 32) showed the most promising anti-cancer activity. The SAR studies pinpointed that the introduction of 4-chloro-phenoxy-*N*-(4-methoxyphenyl)-acetamide substitutions on position 5 of the 4-thiazolidinones ring enhanced the anti-cancer potential^53^.

**Figure 33.**
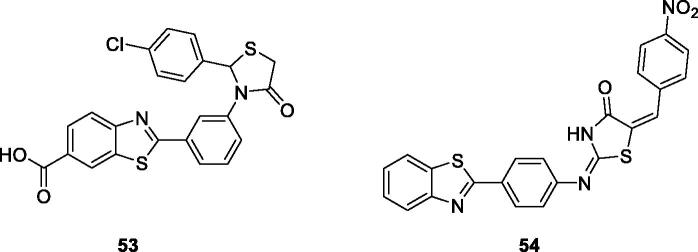
Oxothiazolidine based benzothiazole derivative **53** and thiazolidine benzothiazole derivative **54**.

Prabhu et al. synthesised and studied the anticancer activities of oxothiazolidine based BTA derivatives. The substituted chlorophenyl oxothiazolidine based benzothiazole **53** (Figure 33) showed the most effective anticancer activity against HeLa cell line, inducing 96.8% inhibition and IC_50_ value of 9.76 µM, when compared with the reference drug cisplatin^54^.

Abdelgawad et al. synthesised thiazolidinone containing benzoxazole derivatives by the treatment of aminobenzoic acid with substituted aniline that further reacted in different steps under specific conditions to afford final product and studied their anticancer activities against breast cancer (MCF7) and liver cancer (HEPG2) cell lines. Among all these derivatives, nitrobenzylidene containing thiazolidine derivative **54** (Figure 33) exhibited some anticancer activity, with an IC_50_ value of 36 nM and 48 nM against MCF7 and HEPG2, respectively^55^.

**Figure 34.**
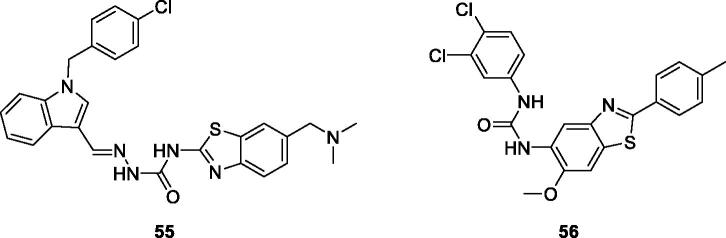
Chlorobenzyl indole semicarbazide BTA derivative **55** and urea BTA derivative **56**.

Ma et al. reported indole based BTA derivatives and studied their anticancer activities. Among all these derivatives, the chlorobenzyl indole semicarbazide benzothiazole **55** (Figure 34) exhibited anticancer activity against four cancer cell lines such as HT-29, H460, A549 and MDA-MB-231. Derivative **55** showed IC_50_ values of 0.024 µM for HT-29, 0.29 µM for H460, 0.84 µM for A549 and 0.88 µM for MDA-MB-231, respectively^56^.

Xie et al. prepared substituted BTA derivatives and studied their anticancer activities. Among all derivatives, the urea benzothiazole **56** (Figure 34) exhibited interesting antitumor activity against 60 cancer cell lines. The average GI_50_ value for derivative **56** was 0.38 µM^57^.

**Figure 35.**
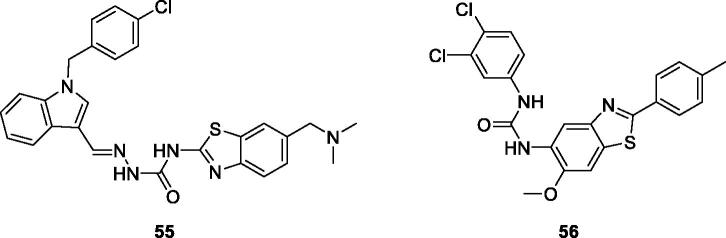
Nitrostyryl containing BTA derivative **57** and fluorostyryl BTA derivative **58**.

Uremis et al. synthesised BTA derivatives using substituted aldehyde with bicyclo[3.2.0]hept-2-en-6-one. The nitro-styryl containing benzothiazole derivative **57** and the fluorostyryl benzothiazole derivative **58** (Figure 35) were reported for their anticancer activity against pancreatic cancer cells having IC_50_ values of 27 ± 0.24 µM for **57** and of 35 ± 0.51 µM for derivative **58**^58^.

**Figure 36.**
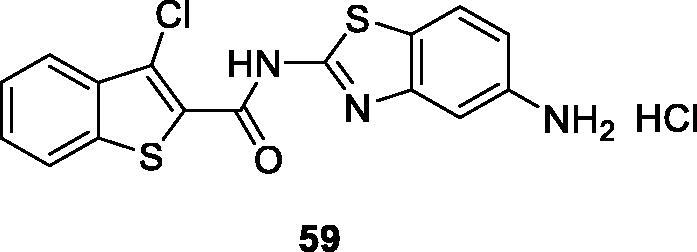
Benzothiophene carboxamide chloroaminobenzothiazole derivative **59**.

Cindric et al. obtained carboxamide containing benzothiazole by the condensation of substituted thiophenes and substituted amino-BTAs to prepare the desired product and studied their anticancer activity against MCF-7 cell line. The benzothiophene based carboxamide chloroaminobenzothiazole **59** (Figure 36) exhibited potent anticancer activity with an IC_50_ of 40 nM^59^.

**Figure 37.**
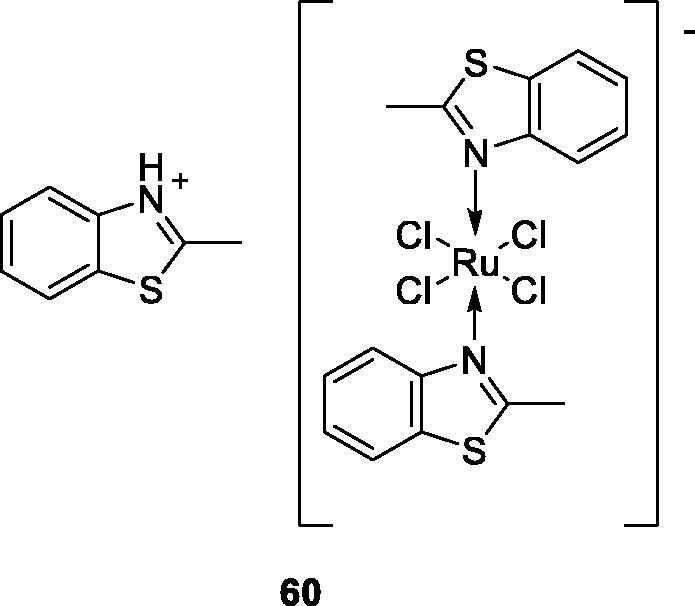
Ru(III) containing methylbenzothiazole derivative **60**.

Nikolova et al. obtained Ru(III) complexes containing benzothiazole derivatives and tested their anticancer activity against K-562 and KE-37 (human leukemic) cell lines. Among all these derivatives, the Ru(III) containing methylbenzothiazole **60** (Figure 37) exhibited the highest cytotoxic activity against K-562 and KE-37, having IC_50_ values of 7.74 ± 2.50 for KE-37 and of 16.21 ± 2.33 for K-562 when compared with the standard drug cisplatin^60^.

**Figure 38.**
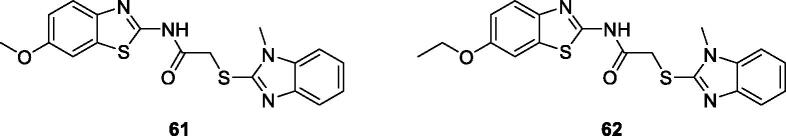
Benzimidazole based acetamide methoxybenzothiazole derivative **61** and acetamide ethoxybenzothiazole derivative **62**.

Yurttas et al. obtained benzothiazole derivatives by acetylation of substituted benzothiazole with chloroacetyl chloride that was further treated in series of reactions to obtain specific derivatives which were tested for their anticancer activities against lung carcinoma (A549) cell line. Derivatives **61** and derivative **62** (Figure 38) exhibited good anticancer activities against A549 with IC_50_ values of 10.67 ± 2.02 µg/mL and 9.0 ± 1.0 µg/mL respectively as compared to the reference compound cisplatin^61^.

**Figure 39.**
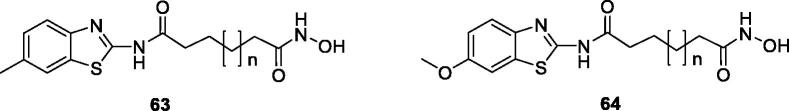
Hydroxamic acid containing methyl/methoxy benzothiazole scaffolds **63** and **64**.

Oanh et al. reported the synthesis of hydroxamic acids containing benzothiazole by the reaction of 2-aminobenzothiazole with adipic acid to produce esters that were converted into the desired BTA hydroxamates, and tested for their anticancer activities against five different cell lines such MCF-7, AsPC-1, SW620, PC3 and NCI-H460. Among the synthesised derivatives, hydroxamic acids **63** and **64** (Figure 39) exhibited good anticancer activities, with the average IC_50_ value of 0.81 µg/mL and 1.28 µg/mL respectively^62^.

**Figure 40.**
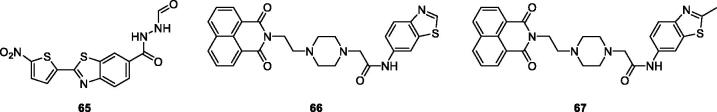
Derivative **65–67** discussed in the article.

Rodrigues et al. synthesised carbohydrazide containing BTA derivatives and studied their antitumor activities against human prostate cancer cell lines. *N*′-formyl-2–(5-nitrothiophen-2-yl)benzothiazole-6-carbohydrazide **65** (Figure 40) exhibited potent anticancer activity against PC-3 and LNCaP having IC_50_ values 19.9 ± 1.17 and 11.2 ± 0.79 µg/m respectively^63^.

Rao et al. reportyed DNA-intercalating naphthalimide-benzothiazole derivatives and evaluated their cytotoxicities against three different cancer cell lines such as HT29, A549 and MCF-7. Among all tested derivatives, the naphthalimide derivative **66** (Figure 40) possessed good antitumor activity against HT-29, A549 and MCF-7 cell lines having IC_50_ values of 3.72 ± 0.3 µM, 4.074 ± 0.3 µM and 07.91 ± 0.4 µM respectively. The naphthalimide **67** (Figure 40) showed antitumor activity with IC_50_ values of 03.47 ± 0.2 µM for HT-29, 03.89 ± 0.3 µM for A549 and 05.08 ± 0.3 µM for MCF-7 cell lines^64^.

**Figure 41.**
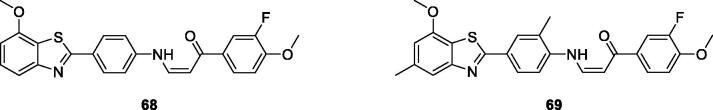
Substituted phenylamino based methoxy benzothiazoles **68** and **69**.

Rao et al. reported the synthesis of 2-arylaminobenzothiazole-arylpropeonones and studied their cytotoxic activities against different human cancer cell lines. Among all synthesised derivatives, substituted phenylamino based methoxybenzothiazole **68** (Figure 41) and substituted phenylamino based methoxy methylbenzothiazole **69** (Figure 41) showed potent cytotoxic activity against HeLa cell lines possessing IC_50_ values of 0.5 ± 0.02 and 0.6 ± 0.29 µM^65^.

**Figure 42.**
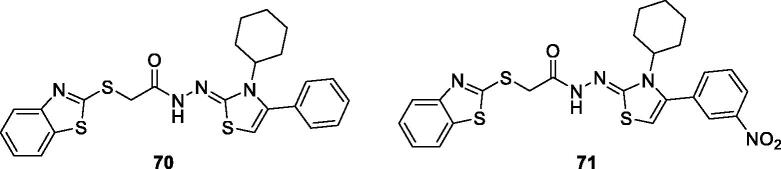
Substituted phenylthizolidene based benzothiazole derivative **70** and **71**.

Osmaniye et al. synthesised benzothiazole-thiazolidine derivatives and studied their anticancer activities against C6 and healthy NIH3T3 cell lines. Among the synthesised derivatives, substituted phenylthizolidene based benzothiazole **70** and substituted nitrophenylthizolidene benzothiazole **71** (Figure 42) exhibited some cytotoxic activities against C6 cell line showing IC_50_ value 0.03 mM. The SAR studies showed that the presence of a phenyl group on the thiazolidine part of the structure increased the selectivity while substitution with an electron withdrawing or donating groups decreased the selectivity^66^.

#### Benzothiazole derivatives with carbonic anhydrase inhibitory and antitumor action

**Figure 43.**
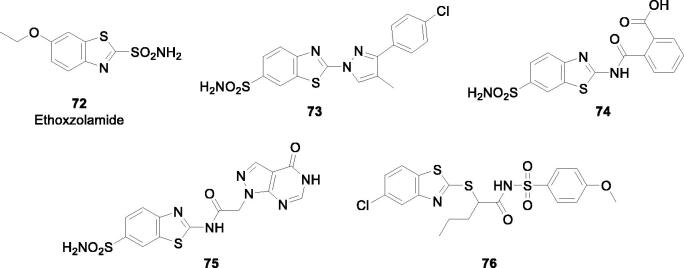
Benzothiazoles with potent CA IX inhibitory action^84–91^.

Carbonic anhydrases (CAs, EC 4.2.1.1) are zinc enzymes which catalyse the reversible interconversion between CO_2_ and bicarbonate^67–71^. CO_2_ is efficiently hydrated through a zinc hydroxide intermediate from the enzyme active site with generation of the weak base bicarbonate and the strong acid H^+^. As a consequence, CAs are involved in pH regulation, electrolyte secretion and metabolism, in normal and tumour tissues^70–72^. Fifteen α-CA isoforms are present in humans, with at least two of tyhem overexporessed in hypoxic tumours (CA IX and XII)^67^^,^^71^^,^^72^, as a consequence of the hypoxia inducible factor (HIF-1α) transcription factor cascade activation^67^^,^^70^. CAs are efficiently inhibited by a range of compounds, such as the sulphonamides and their isosteres^73–76^, and inorganic anions^77^, which constitute the main zinc-binding CA inhibitor (CAI) classes^67^^,^^76^^,^^77^. Some of the CAIs belonging to the sulphonamide^78^, sulfocoumarin^79^, saccharin^80^ or other structurally related chemotypes^81^^,^^82^, were shown to possess significant antitumor effects, with one such derivative (**SLC-0111**) in Phase I/IIb clinical trials for the management of hypoxic metastatic tumours^78^^,^^83^. Indeed, by inhibiting the tumour-associated isoforms CA IX and XII, such CAIs interfere with the pH regulation and metabolism of tumours, leading to the inhibition of growth of the primary tumour, metastases and reducing the population of cancer stem cells^78^. As a consequence, many CAIs belonging to various classes are nowadays investigated for their antitumor/antimetastatic effects, including many BTA derivatives (Figure 43)^84–91^.

.Indeed, using the ethoxzolamide **72**, a CAI in clinical use for decades as lead molecule, a multitude of primary sulphonamides (e.g. compounds **73–75**) as well as the secondary siulfonamide 76 were reported to act as highly efficient, frequently low nanomolar inhibitors against the tumour-associated isoforms CA IX and XII^84–91^. No *ex vivo* or *in vivo* studies are available so far with these potent CA IX/XII inhibitors, but compounds belonging to other classes of sulphonamides were proved to possess significant antitumor effects in vivo when they act as potent inhibitors of these two CA isoforms^78^^,^^92–98^. Thus future studes may address this issue, considering the fact that the BTA scaffold present in these compounds may induce interesting phisico-chemical and pharmacologic properties to the CA IX/XII inhibitors, of which many chemical families are alredy reported^99–103^.

## 4. Conclusions

Benzothiazole is a pharmacophore widely used in medicinal chemistry. This review points out to a growing interest in the development of lead or hybrid structures bearing the BTA moiety as antiproliferative and anticancer agents. The present work describes the potential of BTA scaffolds in the management of various types of cancers such as ovarian, prostate, central nervous system, renal, gastric, pancreatic, liver, breast and colon cancers. SAR studies reaveled that the anticancer activity of BTA scaffolds depends upon the nature of substituents present in these molecules, being multifactorial and not always easy to rationalise. The plethora of research on the anticancer profile of BTA derivatives mentioned in this review and their rationalisation based on the drug targets of these derivatives, when this was possible, may be useful for the development of novel such agents.
